# Editorial

**DOI:** 10.1120/jacmp.v5i2.2033

**Published:** 2004-08-16

With this issue, we are beginning a new procedure for submission and reviews. The Submission Instructions have changed, so please prepare your manuscripts carefully according to the new instructions. The JACMP now uses a double blind review process. This means the reviewers do not know the identity of the author until the paper is accepted and published. We are doing this for several reasons. The double‐blind process is probably the most fair to scholars, as it helps eliminate the bias in favor of well‐known scholars. In addition, if Associate Editors publish in the JACMP, it protect them from the charge the review might not have been sufficiently rigorous and thorough. Many thanks in advance for reviewing our Submission Instructions carefully before contributing your manuscripts.

The JACMP would not be here, except for the support and generosity of our advertisers, and the active participation of our readers. Please show your appreciation by letting them know how much you appreciate their support of the JACMP. In addition, please keep in mind we are counting every use of our banner adds. Every time you visit a vendor through the JACMP, you help the JACMP exist. Unless you use the banner adds, there is no reason for any vendor to stay with us. So whenever you need to visit a vendor, please use the list we provide, or use one of the banner ads. Ultimately, our existence depends on you in so many ways, and this is an easy habit to make.

Michael D. Mills

Editor‐in‐Chief

**The Journal of Applied Clinical Medical Physics**

**Table:** 

**Editorial Board 2004**	
Editor‐in‐Chief	Michael D. Mills
Deputy Editor‐in‐Chief	Timothy D. Solberg
Associate Editor‐at‐Large	Richard Stark
**Associate Editors**	
Radiation Oncology Physics	Nzhde Agazaryan, B. Gino Fallone
	John Gibbons, Michael Herman
	Edwin McCullough, Sasa Mutic
	Timothy Paul, Matthew Podgorsak
	Nikos Papanikolaou
Medical Imaging	Walter Huda, William Pavlicek
Radiation Measurements	Larry DeWerd
Radiation Protection & Regulations	James Deye
Nonionizing Topics	Bhudatt Paliwal
Other Topics	Timothy Solberg
Book/Media Reviews	James Smathers
Editorials	John Horton
**Journal Production Team**	
Layout Editor and Proofreader	Tracy Skelton
Copy Editor	Betty R. Robinson
Manuscript Manager	Patricia Walker

